# Influence of Chest and Diaphragm Manual Therapy on the Spirometry Parameters in Patients with Cerebral Palsy: A Pilot Study

**DOI:** 10.1155/2021/6263973

**Published:** 2021-02-12

**Authors:** Magdalena Rutka, Andrzej Myśliwiec, Tomasz Wolny, Anna Gogola, Paweł Linek

**Affiliations:** ^1^Institute of Physiotherapy and Health Sciences, Musculoskeletal Elastography and Ultrasonography Laboratory, The Jerzy Kukuczka Academy of Physical Education, Katowice, Poland; ^2^Department of Physical Education, The Jerzy Kukuczka Academy of Physical Education, Katowice, Poland; ^3^Institute of Physiotherapy and Health Sciences, Laboratory of Physiotherapy and Physioprevention, The Jerzy Kukuczka Academy of Physical Education, Katowice, Poland; ^4^Department of Physiotherapy, The Jerzy Kukuczka Academy of Physical Education, Katowice, Poland

## Abstract

**Objective:**

To evaluate the influence of manual therapy of the chest and diaphragm on the spirometry parameters in patients with cerebral palsy (CP).

**Method:**

The study was carried out on 20 youths with CP. All participated in 6 sessions (3 sham and 3 actual), with measurements of spirometry at baseline, postsham therapies 1 and 3, before actual therapy, and postactual therapy sessions 1 and 3. Two manual techniques were included: soft tissue mobilization of the chest and the diaphragm.

**Results:**

After the first actual therapy, there was a significant (*p* < 0.01) improvement in forced vital capacity (FVC) by 0.23 L (8% of the average predicted value) and forced expiratory volume in one second (FEV_1_) by 0.18 L (7% of the average predicted value) as compared to results before the therapy. Change in FVC parameter was clinically significant, whereas change in FEV_1_ was not clinically significant. After sham therapy, there was no improvement in spirometry parameters as compared to baseline results.

**Conclusion:**

Single-time manual therapy of the chest and diaphragm has a positive effect on FVC and FEV_1_.

## 1. Introduction

Cerebral palsy (CP) is one of the most common physical disabilities in children. The incidence rate ranges from 1.5 to 3/1000 live births [1]. CP presents many impairments related not only to the neuromotor system but also to the respiratory system [2]. The respiratory system of patients with CP is described in a very general and limited degree [3–5], but it is well-known that patients affected by CP have reduced spirometry parameters [4, 5].

Patients with CP are also characterized by impaired postural control [6]. They exhibit reduction in motor control, lack of trunk stabilization, and progression to muscular atrophy, including respiratory muscles [6, 7]. Know and Lee [4, 5] have suggested that functional motor ability (as classified by the Gross Motor Function Classification System (GMFCS)) [8] and distribution of paralysis could be connected with respiratory function and respiratory muscle weakness in patients with CP. Thus, muscle changes (mainly weakness) and frequent scoliosis contribute to a limitation in physical activity and participation restrictions, which affect pulmonary function in patients with CP [3, 5, 9]. Additionally, patients with CP have restricted chest mobility [9]. As can be seen from previously mentioned studies, patients with CP have many disturbances, which directly or indirectly affect the functioning of the respiratory system.

Until now, proper breathing path, effective cough learning, bronchial tree drainage through body position, chest vibration, or patting are recognized physical therapy treatment methods in patients with CP with reduced vital parameters of the lungs [10]. The literature also presents various forms of active therapy with spirometry devices and during upper extremity resistance exercises [11, 12]. It was confirmed that these therapeutic forms are helpful in the treatment of respiratory dysfunction in patients with CP [11–13], but some of them require conscious participation of the patient and cannot be successfully applied to patients with severe intellectual and motor disabilities. Thus, it is warranted to find other forms of therapy where active patient engagement is not required.

In physical therapy, manual therapy is defined as a clinical approach, including diagnosis and treatment, directed at joint structures and soft tissues [14]. One form of manual therapy is soft tissue mobilization (STM), in which a therapist uses hands-on techniques on muscles, ligaments, or fascia in order to break adhesions and optimize soft tissue function. STM is described as a manual technique employing low-load, long-duration forces applied in approximation, traction, and torsional vectors to improve the mobility between overlying and adjacent connective tissue layers throughout the body [15]. Such techniques are intended to restore the optimal length of the myofascial complex. Manual therapy based on STM can also be conducted without active patient participation and may be potentially useful for patients with severe intellectual and motor disabilities. Heneghan et al. [16] have reported that manual therapy focused on the diaphragm directly improved the functionality of the respiratory system in patients with chronic pulmonary diseases. Similarly, Rocha et al. [17] have reported that manual therapy improves spirometry parameters (e.g., forced vital capacity (FVC)) and chest mobility among patients with chronic obstructive pulmonary disease (COPD). Patients with CP commonly present with restrictive pulmonary disease coupled with reduced chest mobility [6], and this affects the spirometry parameters [18]. Thus, we hypothesized that manual therapy techniques (i.e., STM) focused on the chest and diaphragm may improve spirometry parameters and chest mobility in patients with CP (as in the study by Rocha et al. [17]). To the best of the authors' knowledge, no previous study assessed the potential influence of manual therapy on respiratory parameters in patients with CP.

Due to respiratory consequences associated with CP, such as pneumonia, sleep apnoea, and decreased vital capacity, it seemed important to undertake scientific research that would allow the development of noninvasive and nonpharmacological therapeutic procedures to improve this system [5, 19]. If the function of the respiratory system improves in patients with CP, it will translate into improvement of their quality of life, because respiratory rehabilitation reduces cough and breathing symptoms [20]. This in turn helps reduce social and economic costs due to shortening of the length of hospital stay [21]. Therefore, there is a necessity to conduct further research, which may change existing models of treatment for patients with CP. It may be that manual therapy will help treat and/or prevent respiratory disorders in patients with CP. The objective of this pilot study was to analyse the influence of manual therapy of the chest and diaphragm on spirometry parameters of patients with CP.

## 2. Patients and Methods

Twenty patients with CP aged between 7 and 24 years old were recruited, using convenience sampling. Inclusion criteria were a diagnosis of CP per a neurologist, ability to understand and perform the spirometry instructions, age over 7, and medical clearance from the patient's physician to participate in this study. The exclusion criterion were (a) current systemic disease (e.g., influenza or pneumonia), which could only periodically change the respiratory parameters of the patients [22]; (b) previous chest surgery; and (c) lack of consent. Patients were recruited from three rehabilitation centres in the Silesian region of Poland.

All parents and/or legal guardians and patients received oral and written information about all procedures and provided written informed consent for participation. The attending physician (of qualified patients) clearly stated that there were no contraindications to apply the procedure described in the present research. In addition, the patients were described according to the five-level GMFCS [8].

The study was approved by the Ethics Committee of the Academy of Physical Education, Katowice, Poland, and was conducted in accordance with the guidelines of the Declaration of Helsinki (World Medical Association, 2008).

### 2.1. Blinding Procedures

All spirometric tests were performed by an independent professional technical assistant, who did not know anything about the study experiment. During the whole study, all patients were not aware about their therapy stage: sham or actual.

### 2.2. Measures

**Figure 1:**
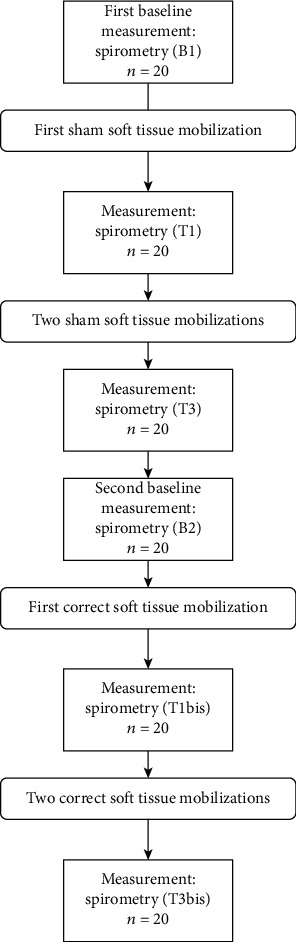
Study flow chart.

The spirometry test was performed six times in the following order (Figure 1): (1) at baseline (B1), (2) after the first (T1) and after the third (T3) simulated sham therapy, (3) two-week break (no spirometry measurements), (4) before the actual therapy (B2), and (4) after the first (T1bis) and third (T3bis) actual STM therapy sessions.

During spirometry measurement, each patient sat on a chair (or in a wheelchair if necessary) with back support and was protected against falling. Before the measurement, the patient took several natural breaths, then (according to the methodology of the device) took a deep maximum inhalation through a single-use mouthpiece and then exhaled the air during a long, strong expiration. The whole measurement was carried out with the air outlet through the nostrils closed. Each test was repeated in order to get three reproducible results, and the best results were selected (even if they came from different expiratory manoeuvres) and used in statistical analyses [23]. The FVC manoeuvre repeatability was considered acceptable when the differences between the largest and the next largest FVC or between the largest and the next largest FEV1 were <0.15 L (if FVC > 1 L) or <0.100 L (if FVC ≤ 1 L) [23]. Between each attempt, the patient had a minute break to exclude the possibility of hyperventilation.

The measurement was carried out with the use of a mobile device, Pony FX (COSMED, Italy), and the following parameters were collected: (a) FVC (L), (b) forced expiratory volume in one second (FEV_1_, L), (c) FEV_1_/FVC (absolute value), and (d) peak expiratory flow (PEF, L/s). The 2012 Global Lung Initiative (GLI) standards were applied to generate the predicted reference values by using original software in the spirometer used.

### 2.3. Interventions

**Figure 2:**
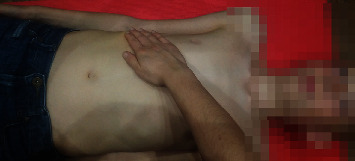
Manual diaphragm release technique.

**Figure 3:**
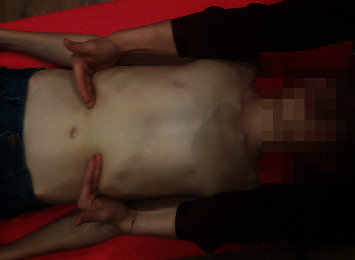
Manual technique release for lower part of the chest. Hand placement for manual diaphragm release technique on the right. Similar hand placement was used on the left side as well.

In the first phase of the study, three (sham) procedures of simulated STM therapy were performed. In this intervention, techniques imitating the target therapy were used, where the touch of the hand was very delicate without using pressure and traction [17]. However, the location of the hands, the duration of the therapy, the number of repetitions, and the duration of the break did not differ between the sham and the actual therapy (detailed description of the techniques used are below). Then, the same patients received a cycle of three actual STM therapies. In both procedures (sham and actual), the therapy was performed at an interval of 2–5 days over a period of 3 weeks. A detailed description of the techniques performed is as follows:

1. The patient was lying in the supine position, with the limbs relaxed, and in the case of patient with contractures, the position was secured by a roller or wedge. The patients received an applied manual therapy (STM) as reported in Figure 2. The physiotherapist made manual contact with the pisiform, hypothenar region, and last three fingers of the hand with the underside of the costal cartilages of the seventh to tenth ribs. During inspiration in the patients, the physiotherapist gently pulled in a cephalad direction, accompanying the elevation movement of the ribs. During expiration, the physiotherapist deepened contact toward the inner costal margin. On subsequent breaths, the therapist sought to gain traction and smoothly deepen the contact [17]. This manoeuvre was performed in 2 cycles of 10 deep inhalations and exhalations (6–10 seconds). There was a one-minute break between cycles [17]
2. The patient was in the supine position. The physiotherapist placed his/her hand on the patient's lower rib arches along their course. Then, he/she gently pressed towards the waist, allowing the hands to slide slightly and slowly together with the subcutaneous tissue, moving layers of deep tissues [24]. The technique was repeated three times at the same speed and force for about 30 seconds during treatment of one side of the body (Figure 3).

### 2.4. Statistical Analysis

Analysis of the collected data was performed using the Statistica 13.1 software package (StatSoft). A one-way analysis of variance (ANOVA) for repeated measurements (within factor was time, baseline data, after first session, sham and actual manual therapies after three sessions, and no between group factor) was used to evaluate the differences in spirometry parameters (FVC, FEV_1_, PEF, and FEV_1_/FVC). For significant differences in the main effect for time, Bonferroni's post hoc test was used. The results are presented as a mean difference and 95% confidence interval (CI). For all analyses, the threshold of the *p* value considered as significant was set at <0.05. In addition to the “statistical significance” approach, a “clinical significance” approach was considered. The latter considers any FEV1 and/or FVC difference by at least 200 mL and/or 12% of the predicted value [25].

## 3. Results

All patients (*n* = 20) completed the entire manual therapy cycle and participated in each of the required tests (Table 1). There was no significant difference in spirometry parameter values between the baseline measurements (B1) and sham therapies (T1, T3). Additionally, there was no significant difference between baseline measurements before sham therapy (B1) and before manual therapy (B2). Detailed results are reported in Table 2.

The analysis reported significant differences in FVC values after the first actual therapy and after the third actual therapy (*p* < 0.001) as compared to the baseline measurement before actual therapy (B2). After the first actual therapy, an improvement in FVC of 0.23 L on average (95% CI: 0.23–0.24) was observed as compared to the baseline results (B2). The average difference in FVC measurements after a series of three treatments was 0.29 L (95% CI: 0.27–0.3) in relation to the baseline results (B2). An increase in FVC of 0.23 L and 0.29 L is statistically (*p* < 0.05) and clinically significant. No difference was found with FVC between the first and third sham manual therapies (T1 and T3, respectively) (Table 2). After the first actual therapy (T1bis), FVC increased by 16% compared to B2 (*p* < 0.001)—this was 8% of the average predicted value. After the third actual therapy (T3bis), FVC increased by 19% compared to the baseline measurement (B2) (*p* < 0.001)—this was 10% of the average reference values. Detailed results are reported in Table 3.

FEV_1_ after the first actual therapy increased on average by 0.18 L (95% CI 0.17–0.21, *p* = 0.04) and after the third actual therapy by 0.24 L (95% CI 0.18–0.31, *p* = 0.005) as compared to the baseline results (B2). An increase in FEV_1_ by 0.18 L is statistically but not clinically significant. After the third therapy, both clinical and statistical increases in FEV1 were observed. No difference was found with regard to FEV_1_ between the first and third sham therapies (T1 and T3, respectively) (Table 2). After the first actual therapy (T1bis), FEV_1_ increased by 16% compared to B2 (*p* = 0.05)—this was 7% of the average predicted value. After the third actual therapy (T3bis), FVC increased by 20% compared to baseline measurement (B2) (*p* = 0.003)—this was 9% of the average reference values. No statistical differences were found for PEF or FEV1/FVC measurements. Detailed results are reported in Table 3.

## 4. Discussion

As a result of the therapy, a significant improvement in FVC and FEV_1_ was noticed after the first actual therapy. The improvements of both parameters (FVC and FEV_1_) were on average 15–16% after the first actual therapeutic intervention. Subsequent actual therapies brought a further increase in the average values of these parameters (FVC and FEV_1_), although they were insignificant in relation to the results obtained after the first actual therapy. After the first and after the third therapy, a statistically and clinically significant improvement of FVC and FEV_1_ parameters was achieved. There were no differences during the sham therapy. Thus, we can suppose that the gained effect after actual therapy was not related to learning or the placebo effect. Per our literature review, this appears to be the first research paper that reports the potential positive effect of manual therapy (based on STM) on respiratory parameters in patients with CP. Therefore, it is not possible to compare the results obtained here with the results of other authors.

From the available literature, we know that regardless of the distribution of paresis and GMFCS classification, patients with CP have significantly reduced values of spirometry parameters [5, 6]. The researchers estimated that, in the group with CP, the respiratory parameters were reduced by 23–67% of the norm [5]. In our study, we found that FVC, FEV_1_, and PEF were reduced by 50–60% as compared to age-, sex-, and height-matched norms. After the third manual therapy, the levels of FVC and FEV_1_ were only reduced by 25–45% as compared to norms. It is possible that the increase in spirometry parameters was caused by changes in the kinematics of the chest and the diaphragm after the implementation of manual therapy. During repeated respiratory cycles, the STM techniques may (a) improve mobility between overlying and adjacent connective tissue layers around the chest, (b) restore the optimal length of the myofascial complex of the chest, (c) mobilize rib cage joints and increase chest wall volume [20], and (d) improve diaphragmatic excursion [26]. Patients with CP have limited chest mobility by shortening of the respiratory muscles and stiffening of the costovertebral joints [11]. Therefore, it is possible that the improvement of the FVC and FEV_1_ parameters after manual therapy of the chest and diaphragm was associated with a change in the mechanics of the chest and respiratory muscles. Such a relationship was reported in COPD patients [17]. Thus, our results should encourage researchers to seek various therapeutic interventions to increase chest mobility, because greater chest expansion is correlated with greater inspiratory lung volume [27].

In order to improve respiratory parameters in patients with CP, researchers tried various therapeutic methods. Some authors have presented a positive effect with upper extremity resistance exercises with elastic bands on maximum respiratory pressure but not on FVC and FEV_1_ spirometry parameters in children with CP [12]. The improvement of spirometry parameters (FEV_1_ and FVC) in patients with CP was noted in another experiment with the use of incentive spirometer exercises [11]. In our study, we also observed positive effects on spirometry parameters but with the use of a different approach than in Young et al. [11]. The programme proposed by Young et al. [11, 27] requires conscious participation of the patient, which will be impossible to implement in more severe forms of CP. This paper has reported the possible positive effect of manual therapy, which does not necessarily require the conscious participation of the patient during the therapy. Thus, manual therapy based on STM can be more widely used in patients with reduced spirometry parameters.

A systematic review by Heneghan et al. [16] tried to evaluate the usefulness of various forms of manual therapy (e.g., osteopathic manipulative therapy, massage, and muscle stretching) in order to improve respiratory function in people with obstructive airway disease, but almost all studies had a high risk of bias with many design/reporting faults. Regardless, manual therapy had a positive effect on lung function [28]. The manual therapy techniques have reported a positive effect on respiratory parameters in another study by Rocha et al. [17], in which STM significantly changed the chest and diaphragm kinematics of patients with COPD. As a result, patients with COPD had increased lung capacity as measured by plethysmography, namely, slow vital capacity and inspiratory capacity [17]. Manual therapy (spinal manipulation) has also been successfully used in children with asthma [29], where an increase in quality of life and a reduction in asthma severity were observed after the intervention. Similarly, Noll et al. [29] have reported that manual therapy (osteopathic manipulation with STM) diminished the duration of antibiotic treatment and reduced the frequency of respiratory failure or death in people with pneumonia. Our study results have reported that, in patients with CP (chronic nature of the disease and disturbed chest mobility in both cases), manual therapy of the chest and diaphragm had positive effects on pulmonary function. Thus, this preliminary study has confirmed that manual therapy orientated on the diaphragm and chest has the potential to be effective in the treatment of patients with CP. Furthermore, even a single session of actual chest and diaphragm therapy had the potential to improve spirometry parameters (FVC and FEV_1_) by 15% in patients with CP. It is also worth noting that manual therapy, as presented here, was as effective in patients with restrictive pulmonary disease (this form occurs commonly in neuromuscular diseases like CP) as compared to patients with COPD. Both forms of pulmonary disease (restrictive and obstructive) have different pathophysiology, but the techniques used were helpful in each lung condition.

In our study, the FEV_1_/FVC ratio and PEF were not changed after the therapy. Unchanged FEV_1_/FVC ratio reflects a restrictive lung condition [30]; thus, the ratio was not expected to change. In restrictive lung conditions (occurring in CP) [4, 5, 13], voluntary lung volumes are restricted due to weakness and/or chest wall tightness; thus, the absolute value of FVC and FEV_1_ will be reduced, but not the relative ratio. In turn, PEF is directly related to the degree of bronchial expansion [31], but the manual therapy used was not dedicated to work on bronchial expansion.

Numerous limitations should also be taken into account in this research. The project cannot be equated with randomized clinical trials (RCTs), although some assumptions of such studies have been preserved (blindness of the patient performing the spirometry test, lack of knowledge about the applied therapy among patients, and use of sham therapy). We decided to perform a number of sessions from the start rather than the RCT because (a) to date, there was no study reporting that manual therapy based on STM is potentially useful for respiratory dysfunction in patients with CP; (b) we have had limited access to patients, and it would be difficult to gather two groups with very similar symptoms in addition to CP; and (c) we wanted to standardize the therapy for all patients and not leave anybody without the therapy at this stage of knowledge about the potential utility of the manual therapy techniques used. Taking into account that we have confirmed the potential usefulness of manual therapy to increase spirometry parameters in patients with CP, there is a reason to prepare RCTs. Another limitation is the study material has quite a significant age range, both sexes, and different degrees/types of functional limitations. Thus, a positive result of manual therapy should be treated as a preliminary one, which requires RCTs on a larger population with a greater number of parameters under consideration (age, sex, classification of GMFCS, and coexisting disorders/restrictions). Additionally, despite the established age criterion, there may be some errors in the results of the spirometry tests associated with the difficulty of performing the test. Lastly, in the conducted studies, individuals performed spirometry tests multiple times; thus, there may have been a learning effect explaining the improvement in the results after the therapy. However, the analyses of results during the first four tests (B1, T1, T3, and B2) have reported that there was no learning effect in this research. The possible effect on the technical assistant, who sees the patient again and again, could also have led to measurement bias.

## 5. Conclusion

Single-time manual therapy of the chest and diaphragm based on STM techniques has a positive effect on FVC and FEV_1_. No positive effect of the therapy on PEF and FEV_1_/FVC ratio was observed. Subsequent therapeutic sessions have caused a slight increase in spirometry parameters. The proposed therapy can be successfully used in the treatment of patients with CP who have disturbances in the respiratory system. Additionally, this study should be the starting point for further studies in which the real effects of manual therapy based on STM on respiratory function in children with CP will be evaluated.

## Figures and Tables

**Table 1:** Clinical characteristics of patient (values are mean ± SD).

Characteristic	Patients
Age (year)	12.9 ± 6.05
Body mass (kg)	47.4 ± 16.6
Height (cm)	149.8 ± 20.4
Body mass index (kg/m^2^)	21.6 ± 3.7
Sex	5 girls/15 boys
GMFCS I	4
GMFCS II	4
GMFCS III	3
GMFCS IV	2
GMFCS V	7

GMFCS: Gross Motor Function Classification System.

**Table 2:** Spirometric parameters before and after sham/actual therapy (mean ± SD).

	Sham therapy			Actual therapy			ANOVA	
	B1	T1	T3	B2	T1bis	T3bis	*F*	*p*
FVC (L)	1.52 ± 1.12	1.56 ± 1.17	1.53 ± 1.17	1.47 ± 1.19	1.70 ± 1.19^∗^	1.76 ± 1.21^∗^	10.25	<0.001
PEF (L)	2.30 ± 1.06	2.32 ± 1.26	2.21 ± 1.28	2.33 ± 1.45	2.53 ± 1.44	2.57 ± 1.41	1.85	0.109
FEV_1_ (L)	1.25 ± 0.84	1.34 ± 1.01	1.26 ± 1.01	1.29 ± 1.01	1.48 ± 1.06^∗^	1.53 ± 1.12^∗^	6.94	<0.001
FEV_1_/FVC (absolute value)	85.9 ± 12.2	86.5 ± 8.96	84.0 ± 12.7	86.9 ± 12.1	84.6 ± 11.0	84.8 ± 785	0.229	0.95

Significant difference in Bonferroni method from ^∗^B2 (*p* < 0.05). B: baseline before sham therapy; T1: after first sham therapy; T3: after third sham therapy; B2: baseline before actual therapy; T1bis: after first actual therapy; T3bis: after third actual therapy; FVC: forced vital capacity; FEV_1_: forced expiratory volume in one second; PEF: peak expiratory flow.

**Table 3:** Spirometric parameter % of predicted value (including age, height, and sex) before and after sham/actual therapy (mean ± SD).

	Sham therapy			Actual therapy			ANOVA	
	B1	T1	T3	B2	T1bis	T3bis	*F*	*p*
FVC (%)	52.2 ± 27.4	53.8 ± 27.4	52.6 ± 27.7	49.2 ± 26.3	57.3 ± 28.2^∗^	58.9 ± 27.7^∗^	7.64	<0.001
PEF (%)	51.1 ± 28.6	50.0 ± 28.9	49.6 ± 34.2	49.3 ± 33.2	51.5 ± 28.2	52.3 ± 27.36	0.26	0.93
FEV_1_ (%)	50.8 ± 25.7	53.9 ± 28.0	51.3 ± 28.6	50.3 ± 25.9	57.7 ± 27.7^∗^	55.9 ± 31.3^∗^	4.03	0.002
FEV_1_/FVC (%)	102.3 ± 14.6	103.0 ± 10.7	95.0 ± 26.8	103.5 ± 14.5	100.7 ± 13.1	68.2 ± 51.9	1.022	0.41

Significant difference in Bonferroni method from ^∗^B2 (*p* < 0.05). B1: baseline before sham therapy; T1: after first sham therapy; T3: after third sham therapy; B2: baseline before actual therapy; T1bis: after first actual therapy; T3bis: after third actual therapy; FVC: forced vital capacity; FEV_1_: forced expiratory volume in one second; PEF: peak expiratory flow.

## Data Availability

Data is available on request.
